# DNA methylation-mediated silencing of matricellular protein dermatopontin promotes hepatocellular carcinoma metastasis by α3β1 integrin-Rho GTPase signaling

**DOI:** 10.18632/oncotarget.2239

**Published:** 2014-07-21

**Authors:** Ying Fu, Ming-Xuan Feng, Jian Yu, Ming-Ze Ma, Xiao-Jin Liu, Jun Li, Xiao-Mei Yang, Ya-Hui Wang, Yan-Li Zhang, Jun-Ping Ao, Feng Xue, Wenxin Qin, Jianren Gu, Qiang Xia, Zhi-Gang Zhang

**Affiliations:** ^1^ State Key Laboratory of Oncogenes and Related Genes, Shanghai Cancer Institute, Renji Hospital, Shanghai Jiao Tong University School of Medicine, Shanghai, China; ^2^ Department of Liver Surgery, Renji Hospital, Shanghai Jiao Tong University School of Medicine, Shanghai, China; ^3^ Department of Plastic Surgery, Shanghai Jiao Tong University Affiliated Sixth People's Hospital, Shanghai, China

**Keywords:** Dermatopontin, Hepatocellular Carcinoma, Patient prognosis, Methylation, Metastasis, α3β1 integrin

## Abstract

Dermatopontin (DPT), a tyrosine-rich, acidic matricellular protein, has been implicated in several human cancers. However, its biological functions and molecular mechanisms in cancer progression, particular hepatocellular carcinoma (HCC), remain unknown. We demonstrated that DPT was significantly down-regulated in 202 HCC clinical samples and that its expression level was closely correlated with cancer metastasis and patient prognosis. The overexpression of DPT dramatically suppressed HCC cell migration *in vitro* and intrahepatic metastasis *in vivo*. We further revealed that the down-regulation of DPT in HCC was due to epigenetic silencing by promoter DNA methylation. And the inhibitory effects of DPT on HCC cell motility were associated with dysregulated focal adhesion assembly, decreased RhoA activity and reduced focal adhesion kinase (FAK) and c-Src tyrosine kinase (Src) phosphorylation, and all of these alterations required the involvement of integrin signaling. Furthermore, we determined that the inhibitory effects of DPT on HCC cell motility were primarily mediated through α3β1 integrin. Our study provides new evidence for epigenetic control of tumor microenvironment, and suggests matricellular protein DPT may serve as a novel prognostic marker and act as a HCC metastasis suppressor.

## INTRODUCTION

Hepatocellular carcinoma (HCC) is a primary malignancy of the liver [1]. Although recent advances have been made in the treatment of HCC, its long-term survival rate is still not satisfactory due to the high rate of recurrence and metastasis after surgery. Accumulating evidence has demonstrated that the tumor microenvironment plays a critical role in the invasiveness and metastasis of cancer [2, 3]. Many molecules that are involved in the interaction between cancer cells and the microenvironment have been proven to affect cancer progression by controlling cancer cell growth, adhesion, migration, invasion and metastasis [4, 5]. Among these molecules, components of the extracellular matrix (ECM) have been shown to have a noticeable impact on metastasis [6, 7].

Dermatopontin (DPT), a tyrosine-rich, acidic ECM protein, was initially isolated from a bovine dermal extract during the purification of decorin [8, 9]. The primary structure of DPT was determined and found to have several important features [8]. One striking feature is its R-G-A-T sequence, which is similar to the integrin-binding R-G-D peptide, indicating that DPT might bind to integrin through this sequence. In fact, it has been shown that DPT binds to α3β1 integrin, as well as a proteoglycan receptor, during cell adhesion [10].

DPT has multiple biological functions in physiological and pathological processes. It accelerates collagen fibrillogenesis [11, 12] and modulates the interaction between decorin and transforming growth factor-β (TGF-β), thereby enhancing the biological activity of TGF-β [13]. The expression of DPT is down-regulated in cutaneous fibrosis [14], hypertrophic scarring, systemic sclerosis [15, 16] and uterine leiomyomas and keloids [17] and may play an important role in wound healing [18, 19]. Recently, DPT was found to be associated with tumor progression. It has been shown that DPT can modulate prostate cell growth *in vivo* [20] and suppress the metastasis of human oral cancer [21]. In addition, DPT was shown to be down-regulated in hepatocellular carcinoma [22]. However, the functional characterization and molecular mechanism by which DPT affects tumor progression, particularly for HCC, has not yet been fully explored.

In this study, we evaluated the expression status, clinical relevance and functional role of DPT in HCC. We first examined DPT expression in 202 HCC samples by immunohistochemical staining and found that DPT expression was significantly down-regulated and was closely related to indicators of tumor metastasis, such as vascular invasion and tumor thrombosis, and to patient prognosis. We then demonstrated that DPT suppressed HCC cell proliferation and motility *in vitro* and HCC growth and metastasis *in vivo*. Meanwhile, we found that the down-regulation of DPT in HCC was mainly mediated by DNA methylation. Furthermore, we uncovered that the inhibitory effects of DPT on HCC motility were primarily mediated by α3β1 integrin-Rho GTPase signaling. Thus, we demonstrated, for the first time, the role of DPT in HCC metastasis.

## RESULTS

### DPT expression is down-regulated in HCC and closely related to vascular invasion and patient prognosis

**Figure 1:**
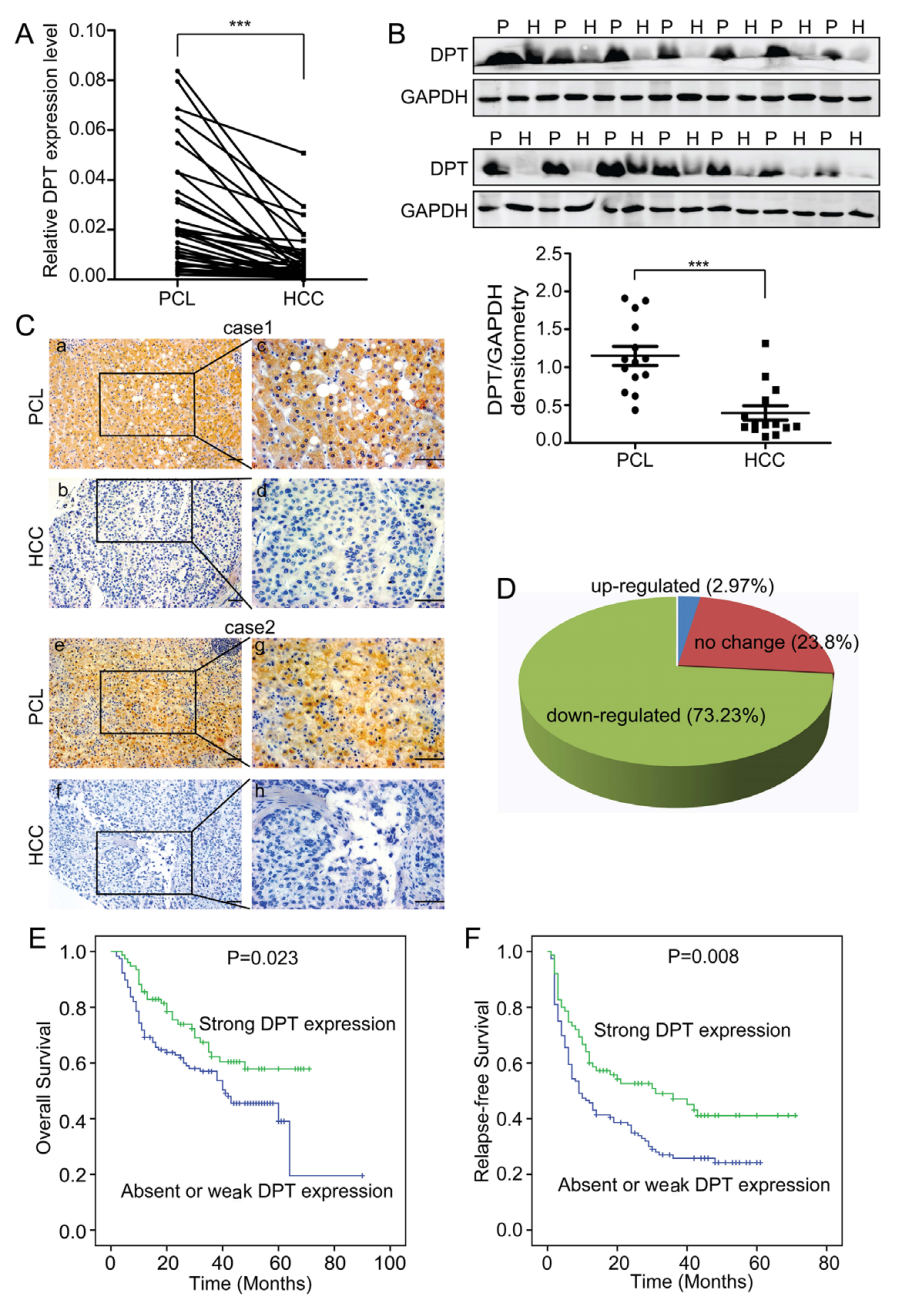
DPT is down-regulated and closely related to patient prognosis in hepatocellular carcinoma (HCC) (A) Relative mRNA expression of DPT, as determined by qPCR, in 36 pairs of HCC tissues and their paracancerous liver (PCL) tissues. Values are means ± SEM (*** *P* < 0.001). (B) Western blotting analysis of DPT expression in 14 pairs of HCC and PCL. P represents paracancerous liver tissues and H represents HCC tissues. The densitometric analysis of the results was shown below. Glyceraldehyde-3-phosphate dehydrogenase (GAPDH) was included as a loading control. Values are means ± SEM (*** *P* < 0.001). (C) Immunohistochemical staining of DPT in HCC and PCL (Original magnification: a, b, e and f, 200x; c, d, g and h, 400x). Scale bars, 10μm. (D) The expression of DPT was down-regulated in 73.23% of the HCC patients. n = 168. (E) Kaplan-Meier analysis of overall survival for the expression of DPT (*P =* 0.023). (F) Kaplan-Meier analysis of relapse-free survival for the expression of DPT (*P =* 0.008).

To compare the expression levels of DPT in HCC tissues and their paracancerous liver (PCL) tissues, we performed quantitative real-time polymerase chain reaction (qPCR) for 36 pairs of HCC/PCL tissues and western blotting for 14 pairs of HCC/PCL tissues. The results showed that the DPT expression level was significantly lower in the HCC tissues than in the PCL tissues (Figure 1A and 1B). We further evaluated the expression of DPT in 202 paired HCC and PCL tissues by immunohistochemical staining. Stronger DPT staining was detected in the PCL tissues than in the HCC tissues (Figure 1C). The expression level of DPT was down-regulated in 73.2% of the HCC patients (Figure 1D).

To further investigate the clinical significance of DPT in HCC, we examined the correlation between the DPT expression status and clinicopathological characteristics of 202 HCC patients who were divided into two groups: the high expression group (n = 65) and the low expression group (n = 137). The results indicated that the expression level of DPT in the HCC tissues was closely associated with vascular invasion (*P =* 0.005), tumor thrombosis (*P =* 0.003), tumor encapsulation (*P =* 0.013), tumor differentiation (*P =* 0.017), serum AFP (*P =* 0.016) and TNM stage (*P =* 0.047) (Table 1). We then analyzed the correlation between DPT protein expression and patient prognosis and found that the patients with strong DPT expression had higher rates of overall survival (OS) (*P =* 0.023) and relapse-free survival (RFS) (*P =* 0.008) than patients with absent or weak DPT expression (Figure 1E and 1F). Taken together, these data strongly imply that DPT has an inhibitory effect on HCC metastasis and acts as an indicator of HCC metastasis and prognosis.

**Table 1:** Clinicopathological correlation of DPT expression in 202 HCC patients using Pearson's x^2^ test

Variable		DPT (n)		
		High, n (%)	Low, n (%)	*P*
Age	≤50 years	23 (11.39)	81 (40.10)	0.184
	>50 years	42 (20.79)	56 (27.72)	
Gender	Female	8 (3.96)	20 (9.90)	0.660
	Male	57 (28.22)	117 (57.92)	
Liver Cirrhosis	Yes	56 (27.72)	121 (59.90)	0.662
	No	9 (4.46)	16 (7.92)	
Local	Yes	9 (4.50)	13 (6.50)	0.372
	No	56 (28.00)	122 (61.00)	
Tumor multiplicity	Single	56 (27.72)	112 (55.45)	0.435
	Multiple	9 (4.45)	25 (12.38)	
Serum AFP	≤20ng/ml	30 (15.00)	39 (19.50)	0.016
	>20ng/ml	35 (17.50)	96 (48.00)	
Tumor satellite	Yes	7 (3.48)	25(12.44)	0.168
	No	58 (28.86)	111(55.22)	
Tumor encapsulation	None	27 (13.43)	82 (40.80)	0.013
	Complete	38 (18.90)	54 (26.87)	
Thromb	Yes	6 (2.97)	38 (18.81)	0.003
	No	59 (29.21)	99 (49.01)	
Tumor differentiation	I	1 (0.50)	2 (0.99)	0.017
	II	32 (15.92)	40 (19.90)	
	III	32 (15.92)	94 (46.77)	
Vascular invasion	Yes	10 (4.95)	47 (23.27)	0.005
	No	55 (27.23)	90 (44.55)	
Tumor size	≤5 cm	37 (18.32)	65 (32.18)	0.208
	>5 cm	28 (13.86)	72 (35.64)	
TNM stage	I	46 (22.77)	73 (36.14)	0.047
	II	8 (3.96)	15 (7.43)	
	III	11 (5.44)	49 (24.26)	

Tumor differentiation: Tumors were divided into three groups according to the Edmondson–Steiner classification. Grade I: well differentiated tumors. Grade II: moderately differentiated tumors. Grade III: poorly differentiated tumors.

TNM stage: HCC tumors were classified according to the staging system by the International Union against Cancer (UICC) as follows: Stage I, encapsulated and without evidence of liver or vascular invasion; Stage II, unencapsulated or capsulated and with liver invasion but without vascular invasion; Stage III, invasion of small vessels in the tumor capsule or focal invasion of portal vein branches close to the tumor or invasion of the portal vein in distal the liver, branches of the major portal vein, and the common bile duct or perforation into visceral peritoneum.

### Detailed characterization of DPT expression in normal liver, PCL, HCC and thrombus tissues from the same individual patients

**Figure 2:**
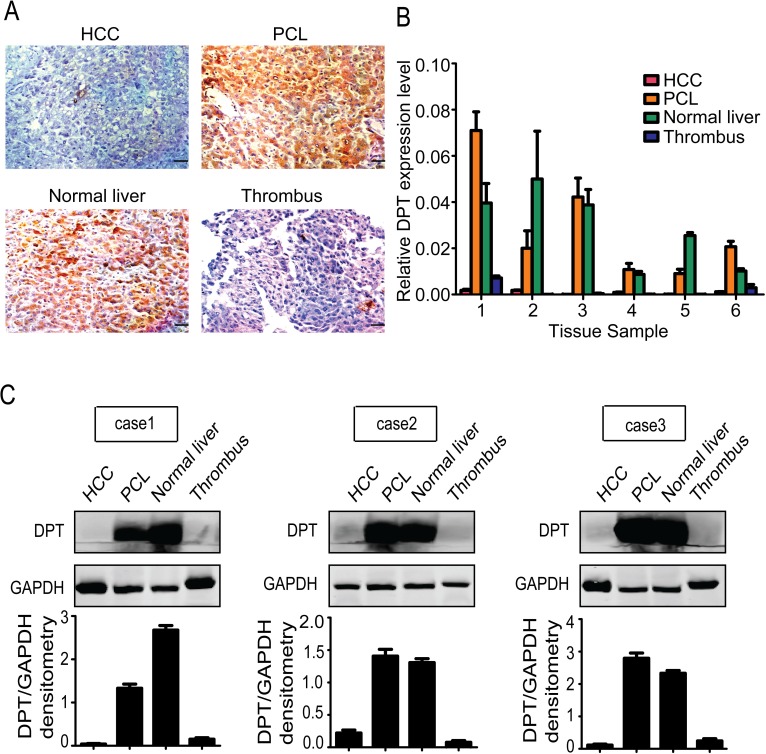
Detailed characterization of DPT expression in normal liver, paracancerous liver (PCL), hepatocellular carcinoma (HCC) and thrombus tissues from the same individual patients (n=6) (A) Immunohistochemical staining of DPT in HCC, PCL, normal liver and thrombus tissues. Scale bars, 10 μm. (B) Relative mRNA expression of DPT in HCC, PCL, normal liver and thrombus tissues. (C) Western blotting analysis of DPT expression in HCC, PCL, normal liver and thrombus tissues. Values were normalized to GAPDH.

To further assess the relationship between the expression level of DPT and HCC metastasis, we collected normal liver, PCL, HCC and tumor thrombus tissues from the same individual patients and performed immunohistochemical staining. A higher DPT expression level was detected in the normal liver and PCL tissues compared to the HCC and tumor thrombus tissues (Figure 2A), which was confirmed by qPCR (Figure 2B) and western blotting (Figure 2C). These data further indicate that DPT may play an important role in modulating the invasiveness and metastasis of HCC.

### DPT is epigenetically silenced by promoter DNA methylation

**Figure 3:**
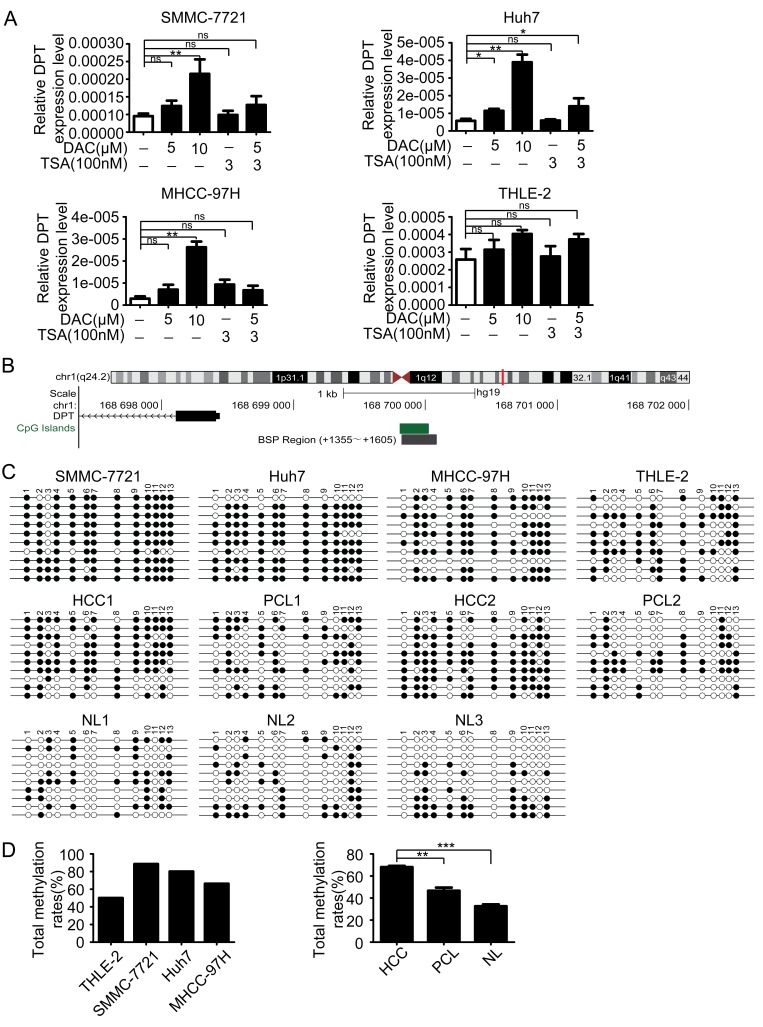
DPT is down-regulated by hypermethylation of DNA promoter (A) The reexpression of DPT was evaluated by qPCR in the HCC cell lines and THLE-2 cell line treated with no drug, DAC, TSA or DAC plus TSA. 18s RNA was used as a loading control. Values are means ± SEM (*P < 0.05, **P < 0.01), ns means no significance. (B) Schematic representation of the location of DPT and CpG island within the promoter in chromosome and the primers designed for bisulfite sequencing. (C) Bisulfite-sequencing results of three HCC cell lines (SMMC-7721, Huh7 and MHCC-97H), THLE-2 cell line, 2 pairs of HCC and their paracancerous liver (PCL) tissues and 3 normal liver (NL) tissues. All 13 CpG sites were sequenced. Open circles indicate unmethylated and solid circles represent methylated CpG dinucleotides. (D) The total methylation rates of DPT promoter in the three HCC cell lines (SMMC-7721, Huh7 and MHCC-97H) and THLE-2 cell line, and in HCC, PCL, NL tissues. Values are means ± SEM (**P < 0.01, *** *P* < 0.001).

During our preliminary studies, we found that DPT was significantly down-regulated in HCC. Similarly, DPT expression level has a prominent decrease in human oral squamous cell carcinoma (OSCC) [23]. However, the regulatory mechanism underlying DPT down-regulation in HCC is still unclear. Epigenetic modification including DNA methylation and histone modification are considered as the main regulatory mechanisms involved gene aberrant expression [24]. To explore the epigenetic regulation of DPT expression in HCC, three HCC cell lines SMMC-7721, Huh7 and MHCC-97H and an immortalized human liver cell line THLE-2, were treated with a demethylating agent 5-aza-2′-deoxycytidine (DAC) and a histone deacetylase inhibitor trichostatin A (TSA) both separately and in conjunction. The results showed that the expression level of DPT in HCC cells was significantly increased by treatment with DAC in all tested HCC cell lines, but not by treatment with TSA. While the expression level of DPT in THLE-2 cells was only slightly increased by treatment with DAC (Figure 3A).

To further confirm the methylation-mediated silencing of DPT expression in HCC, we analyzed genomic structure of DPT and found there was a CpG island in DPT promoter region (Figure 3B). We then performed bisulfite sequencing PCR (BSP) for SMMC-7721, Huh7, MHCC-97H, THLE-2 cells and 5 pairs of HCC and their paracancerous liver (PCL) tissues and other 3 normal liver (NL) tissues. The methylation status of every CpG site in DPT promoter of these cell lines and tissues were shown in Figure 3C and Supplementary Figure 1. The results revealed that the HCC cells had higher methylation rates of DPT promoter compared with normal human liver cells. Furthermore, DPT was also hypermethylated in HCC tissues than those in PCL and normal liver tissues (Figure 3D).

### DPT suppresses HCC cell proliferation *in vitro* and tumor growth *in vivo*

To explore the biological functions of DPT in HCC progression, we established DPT-overexpressing stable cell lines, which were transfected with a lentivirus carrying the DPT gene and labeled as Lenti-DPT, using SMMC-7721 and Huh7 cells. Meanwhile, control cells were transfected with a mock vector and designated as Lenti-vector. DPT overexpression in these two HCC cell lines was confirmed by qPCR and western blotting (Supplementary Figure 2A and 2B).

**Figure 4:**
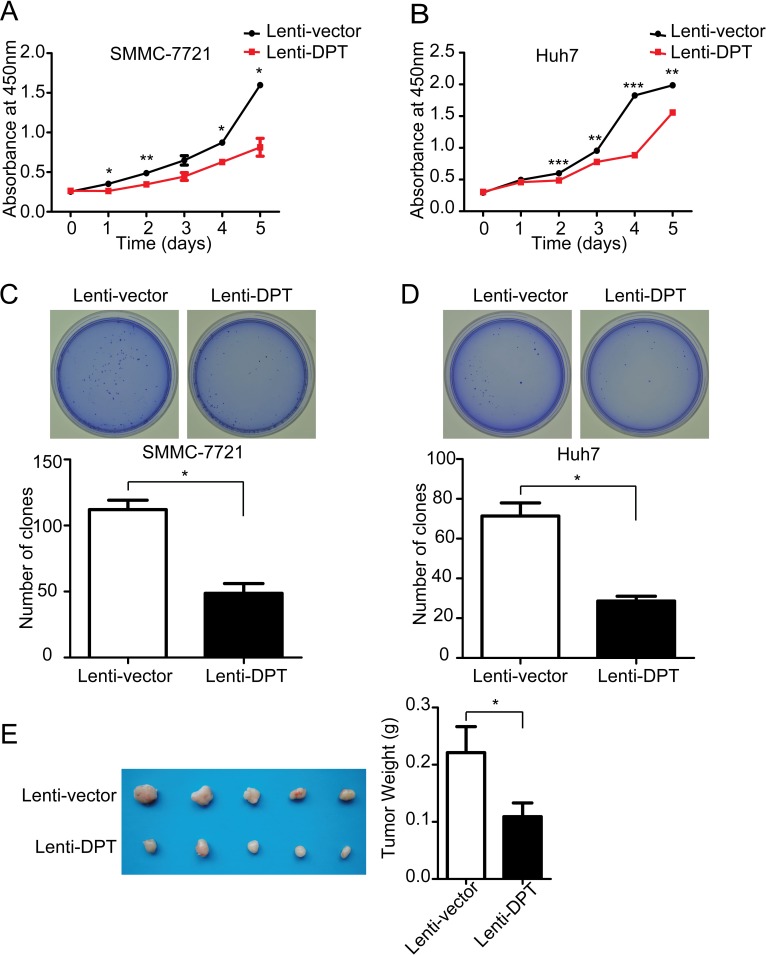
DPT overexpression suppresses HCC cell proliferation *in vitro* and tumor growth *in vivo* (A) Cell proliferation analysis of Lenti-vector/SMMC-7721 and Lenti-DPT/SMMC-7721 cells, as measured by CCK-8 assay. n = 6. Values are means ± SEM (*P < 0.05, **P < 0.01). (B) Cell proliferation analysis of Lenti-vector/Huh7 cells and Lenti-DPT/Huh7 cells, as measured by CCK-8 assay. n = 6. Values are means ± SEM (**P < 0.01, *** *P* < 0.001). (C-D) The representative dishes show the colony formation results of DPT overexpression on SMMC-7721 (C) and Huh7 (D) cells. Statistical analysis of the colonies number was made from 3 independent experiments (means ± SEM, *P < 0.05). (E) Morphologic characteristics of tumors (left) and tumor weight analysis (right) of Lenti-vector/SMMC-7721-inoculated mice and Lenti-DPT/SMMC-7721-inoculated mice sacrificed at six weeks. n = 5. The results shown are means ± SEM (*P < 0.05).

We first examined the effect of DPT overexpression on HCC cell growth. The results showed that the overexpression of DPT significantly suppressed the proliferation of the SMMC-7721 and Huh7 cells *in vitro* by Cell Counting Kit-8 (CCK8) assay (Figure 4A and 4B) and soft agar colony formation (Figure 4C and 4D). To further confirm the results *in vivo*, Lenti-vector or Lenti-DPT cells (SMMC-7721) were subcutaneously inoculated into nude mice. The tumors derived from the Lenti-DPT cells were significantly smaller than those derived from the Lenti-vector cells (Figure 4E).

### DPT reduces HCC cell migration and invasion *in vitro* and metastasis *in vivo*

**Figure 5:**
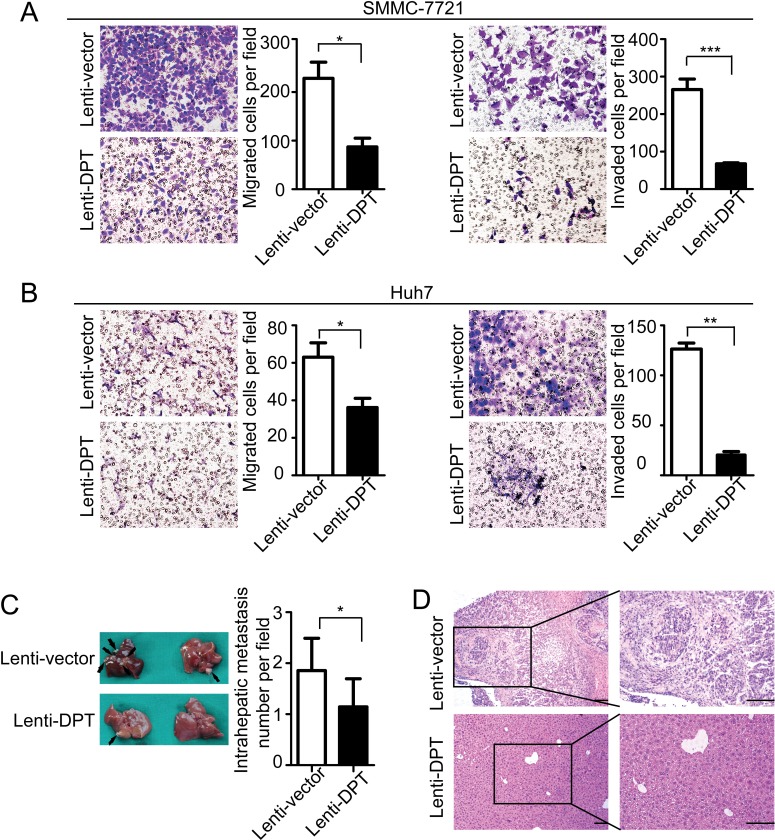
DPT overexpression reduces HCC cell migration and invasion *in vitro* and inhibits intrahepatic metastasis *in vivo* (A-B) Representative images of cellular migration and invasiveness of Lenti-vector/SMMC-7721, Lenti-DPT/SMMC-7721 (A), Lenti-vector/Huh7 and Lenti-DPT/Huh7 (B) cells are shown. Quantification of migrated and invaded cells were performed for six randomly selected fields (original magnification: 200x). (C) Representative photographs of intrahepatic metastases in Lenti-DPT/SMMC-7721-inoculated mice and vector control mice, along with the number of metastatic nodules in the livers, are shown. Black arrows indicate metastases. (D) Representative images of H&E staining of the liver tissues from (C).

**Figure 6:**
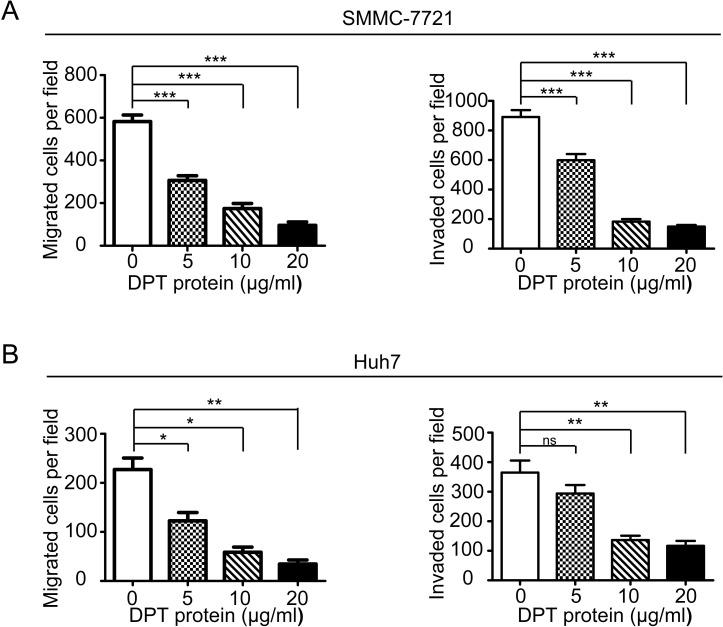
Recombinant human DPT suppresses SMMC-7721 and Huh7 cells migration and invasion *in vitro* (A-B) SMMC-7721(A) and Huh7 (B) cells were performed migration and invasion assays by treating with 0, 5, 10 or 20μg/ml recombinant human DPT. Migrated or invaded cells were counted from six randomly selected fields (original magnification: 200 x). The results are shown as means ± SEM (*P < 0.05, **P < 0.01, *** *P* < 0.001).

To further explore the functional role of DPT in HCC metastasis, we first investigated the effects of DPT on HCC cell migration and invasion *in vitro*. The results showed that the overexpression of DPT significantly inhibited SMMC-7721 (Figure 5A) and Huh7 (Figure 5B) cell migration and invasion *in vitro*. Because DPT is an extracellular matrix protein, we further confirmed the suppressive role of DPT on HCC cell migration and invasion by using recombinant human DPT protein. The results showed that the recombinant DPT protein significantly inhibited SMMC-7721 and Huh7 cell migration and invasion in a dose-dependent manner (Figure 6 and Supplementary Figure 3).

To examine the role of DPT in HCC metastasis *in vivo*, using a microsyringe, the left hepatic lobes of nude mice were orthotopically inoculated with Lenti-DPT/SMMC-7721 or Lenti-vector/SMMC-7721 cells. Six weeks after the liver implantation, the mice were sacrificed and their livers were examined. The number of intrahepatic metastatic nodules was much lower in the mice inoculated with the Lenti-DPT/SMMC-7721 cells than in the mice inoculated with the Lenti-vector/SMMC-7721 cells (Figure 5C). Histological examination of the liver tissue also showed that DPT overexpression suppressed the metastatic potential of HCC *in vivo* (Figure 5D).

### Dysregulation of focal adhesion assembly and Rho GTPase signaling by DPT overexpression

**Figure 7:**
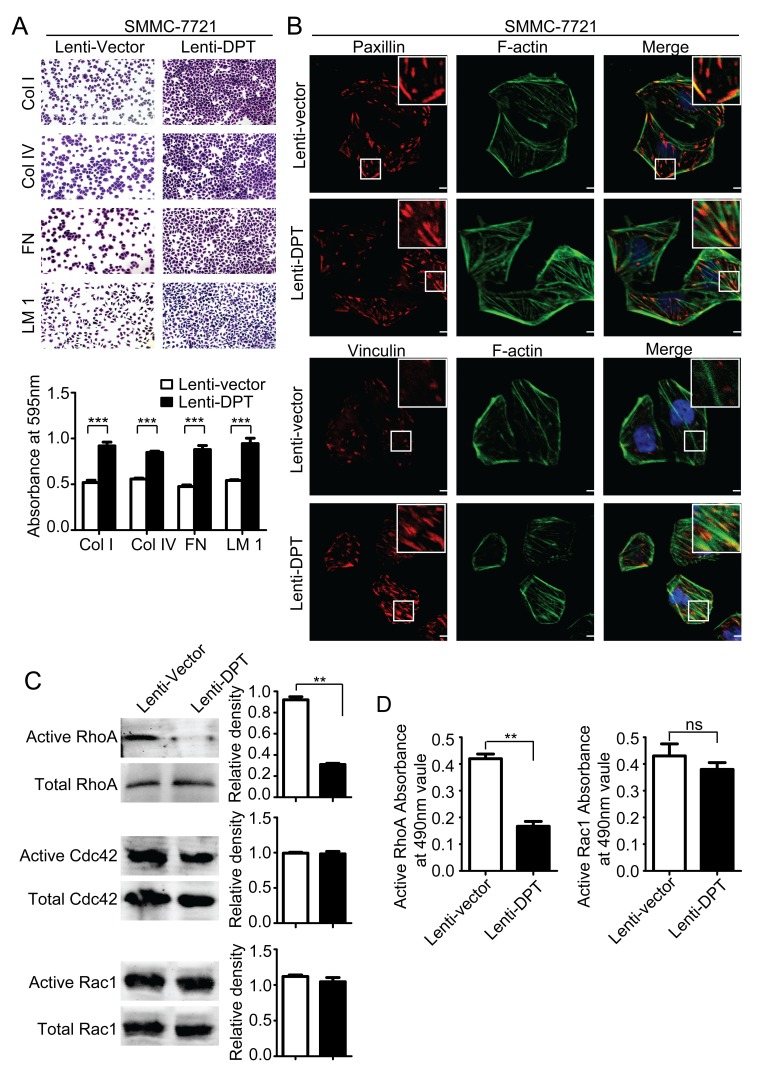
Overexpression of DPT enhances SMMC-7721 cell adhesion and affects focal adhesions and the activity of Rho GTPase (A) DPT overexpression increased the adhesion of SMMC-7721 cells to collagenI (ColI), collagenIV (ColIV), fibronectin (FN) and laminin1 (LM1). Adherent cells were stained with crystal violet and quantified by colorimetry (***P < 0.001). (B) The distribution of paxillin (upper panel) and vinculin (lower panel) was analyzed by immunofluorescence. Red fluorescence represents paxillin or vinculin staining. F-actin is shown by green fluorescence, and the cell nuclei were stained with DAPI (blue fluorescence). Scale bars, 10μm. (C) Analysis of the active and total RhoA, Cdc42 and Rac1 in Lenti-vector/SMMC-7721 and Lenti-DPT/SMMC-7721 cell lysates by pull-down assay. Values are means ± SEM (**P < 0.01). (D) The G-LISA results of active RhoA and Rac1. Values are means ± SEM (**P < 0.01), ns means no significance.

It has been reported that DPT enhances the adhesion of fibroblasts and epidermal keratinocytes via α3β1 integrin [10, 25, 26]. Therefore, we analyzed the adhesive capacity of the Lenti-DPT/SMMC-7721 and Lenti-vector/SMMC-7721 cells and found that overexpression of DPT significantly enhanced the adhesion of SMMC-7721 cells to the most common ECM proteins, such as collagen, laminin and fibronectin (Figure 7A). Integrin-mediated cell-matrix adhesion plays an important role in the regulation of cell migration [27]. This process is always accompanied with the transformation of focal adhesions, which are composed of a variety of molecules, including regulatory molecules and adapter proteins [28]. Among these molecules, paxillin is a marker of newly formed focal adhesions, and vinculin has been demonstrated to be a key player in the maturation and stability of focal adhesions [29]. Next, we investigated focal adhesion assembly in the Lenti-DPT/SMMC-7721 and Lenti-vector/SMMC-7721 cells by immunofluorescent staining of these two adapter proteins, vinculin and paxillin. As shown in Figure 7B, compared to the Lenti-vector/SMMC-7721 cells, there were many more vinculin-containing focal adhesions in the Lenti-DPT/SMMC-7721 cells, and they were distributed not only at the periphery but also at the ventral surface of the spread cells. No obvious differences were observed in the number or localization of the paxillin-containing focal adhesions between the Lenti-vector/SMMC-7721 and Lenti-DPT/SMMC-7721 cells.

Small Rho GTPases are key molecules in the regulation of focal adhesion formation and are associated with actin stress fibers [30]. Additionally, the activity of Rho GTPases affects the locomotion of tumor cells [31]. Thus, we examined Rho GTPase activity in the Lenti-DPT/SMMC-7721 and Lenti-vector/SMMC-7721 cells. Our pull-down assay results revealed that the overexpression of DPT reduced the activity of RhoA but had no effect on the activity of Cdc42 or Rac1 (Figure 7C). To further confirm the effect of DPT on Rho GTPases activities, we performed G-LISA assay, another approach to measure the activities of Rho GTPases for RhoA and Rac1. The G-LISA results also revealed that the overexpression of DPT significantly reduced the activity of RhoA, but it had no effect on Rac1 activity (Figure 7D).

Taken together, these DPT-induced alterations, including enhanced focal adhesion stability, reduced small Rho GTPase activity and decreased cell motility, indicate the involvement of integrin signaling in the activity of DPT in SMMC-7721 cells.

### Silencing of α3 integrin reverses DPT-suppressed HCC motility and integrin-associated signaling

**Figure 8:**
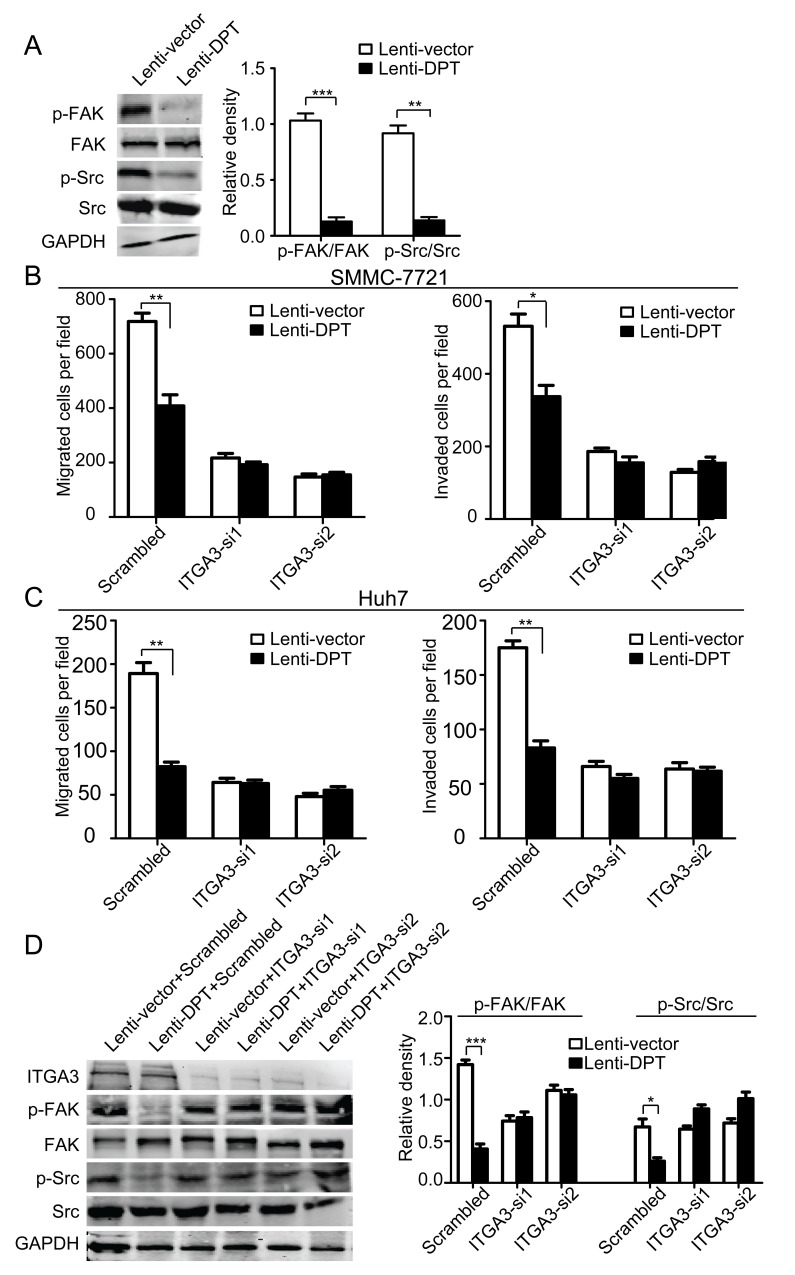
Knockdown of α3 integrin by RNA interference abrogates the attenuated migration and invasion of HCC cells and the modification of integrin-associated signaling caused by DPT overexpression (A) Western blotting analysis of FAK and Src phosphorylation in DPT-overexpressing and control SMMC-7721 cells. The relative quantification of FAK and Src phosphorylation is shown in the right panel. GAPDH was used as the loading control. Values are means ± SEM (**P < 0.01, *** *P* < 0.001). (B-C) Lenti-vector/SMMC-7721, Lenti-DPT/SMMC-7721 (B), Lenti-vector/Huh7 and Lenti-DPT/Huh7 cells (C) were transfected with siRNAs against α3 integrin, and migration and matrigel invasion assays were performed. The results showed that the difference in the number of migrated and invaded cells caused by DPT overexpression disappeared after the silencing of α3 integrin. Values are means ± SEM (*P < 0.05, **P < 0.01). (D) Western blotting analysis of FAK and Src phosphorylation in DPT-overexpressing and control SMMC-7721 cells transfected with scrambled or ITGA3-siRNA. The densitometric analysis of FAK and Src phosphorylation is shown in the right panel. GAPDH was used as the loading control. Values are means ± SEM (*P < 0.05, ***P < 0.001).

Integrins are the major receptors for ECM proteins. To investigate whether integrins or their associated signaling molecules are involved in the DPT-induced suppression of HCC migration, we first examined the phosphorylation of FAK and Src, two important integrin-associated signaling molecules. The results showed that the overexpression of DPT significantly reduced the phosphorylation levels of FAK and Src (Figure 8A). It has been reported that DPT binds to α3β1 integrin [10]. Thus, we further explored whether the inhibitory effects of DPT on HCC motility were mediated by α3β1 integrin. To confirm our hypothesis, α3β1 integrin was silenced by the application of small interfering RNA (siRNA). Using a migration and invasion assays, we found that the inhibitory effects of DPT on HCC migration and invasion were almost completely abolished by the silencing of α3 integrin in both SMMC-7721 (Figure 8B) and Huh7 cells (Figure 8C). The decreased phosphorylation levels of FAK and Src caused by DPT overexpression were also reversed by α3 integrin knockdown (Figure 8D). These data indicate that the inhibitory effects of DPT on HCC motility are primarily mediated through α3β1 integrin.

## DISCUSSION

Postoperative recurrence and metastasis are the leading causes of death for HCC patients [32, 33]. Therefore, it is extremely crucial to explore the molecular mechanism underlying HCC metastasis. The ECM has been shown to play an important role in the processes of tumor invasion and metastasis [28, 34]. DPT, a matricellular protein, has been reported to have putative functions in cell–matrix interactions and matrix assembly. In this study, we examined the expression of DPT in HCC tissues and their paracancerous liver tissues and demonstrated that DPT was significantly down-regulated in HCC patients. By analyzing the prognosis of HCC patients, we further demonstrated that the expression level of DPT was closely correlated with overall survival and relapse-free survival in HCC. Therefore, our results suggest that DPT may act as a prognostic factor for HCC.

A previous study suggested DPT as a metastatic marker of human oral cancer [21]. Nevertheless, the biological functions and molecular mechanisms of DPT in cancer metastasis have not been investigated. In this study, we demonstrated for the first time that DPT suppressed HCC metastasis via α3β1 integrin-Rho GTPase signaling. It is well known that ECM proteins function mainly through interactions with cell surface receptors, such as integrins, syndecans, etc. [35] Among these molecules, integrins are the major ECM receptors, and they play an important role in tumor progression, especially tumor invasion and metastasis [36]. In response to extracellular ligand binding, integrins undergo a conformational change that permits the recruitment of cytoplasmic adaptor proteins and signaling molecules [37], including small Rho GTPases, Cdc42, Rac1 and RhoA, which are key players in cell migration and cancer metastasis.

Small Rho GTPases mainly affect the cell movement and tumor metastasis by regulating the formation of focal adhesion and cytoskeleton reorganization. Especially RhoA, plays an important role in controlling the assembly of actin stress fibers to generate contractile forces and then influences cell movement. Recent studies found that recurrent RhoA mutations occur in diffuse-type gastric carcinoma and mutant RhoA worked in a gain-of-function manner [38, 39]. In this study, we demonstrated that DPT, through its interaction with α3β1 integrin, reduced the phosphorylation levels of FAK and Src, decreased the activation of RhoA and enhanced the maturation and stability of focal adhesions, resulting in the suppression of HCC cell motility.

It has been reported that DPT competes with decorin to interact with TGF-β and enhance its biological activity [13]. TGF-β, one of the most important cytokines in physiological and pathological processes, has been shown to have multiple functions in tumor progression, such as in the regulation of growth arrest and apoptosis and tumor suppression [40]. In this study, we found that the overexpression of DPT suppressed HCC cell proliferation *in vitro* and *in vivo*. However, whether the inhibitory effects of DPT on HCC cell growth are related to TGF-β signaling requires further investigation. The latest evidence suggests that there is a cross-talk among TGF-β signaling molecules, integrins and the extracellular matrix [41, 42]. Therefore, we hypothesized that α3β1 integrin and TGF-β had a synergistic effect on DPT in the control of HCC progression.

Dysregulated gene expression, which caused by aberrant DNA methylation, contributes to the progression of several human cancers including HCC [43]. In this study, we found that DPT was significantly down-regulated in HCC and facilitated to HCC metastasis. This finding is in accordance with previous reports in that the expression of DPT is decreased in some pathological processes, such as cutaneous fibrosis [14], hypertrophic scarring, systemic sclerosis [15, 16], uterine leiomyomas [17] and hepatocellular carcinoma [22]. More importantly, we revealed for the first time that DPT was epigenetic silenced by the aberrant hypermethylation of CpG islands in promoter in HCC. The same epigenetic regulation may also exsit in other reported pathological processes and it is worthy of further in-depth study. Additionally, the DPT expression can be elevated in HCC cells by treatment with the demethylating agent DAC. And recent studies shed light on the epigenetic therapy including numerous drugs that target specific enzymes involved in the epigenetic regulation of gene expression for cancers [44]. Therefore, together with our research data prompted that utilizing demethylating agents to enhance DPT expression may have a potential value for HCC treatment.

It is generally considered that, during carcinogenesis processes, epigenetic modifications of the genome, including DNA methylation and histone modification, are established as a consequence of interactions between cellular lineage and environmental input. However, increasing evidence indicated that epigenetic changes were responsible for initiation, proliferation, invasion and metastasis of tumor cells by affecting tumor microenvironment [45, 46]. For example, some ECM proteins including tumor suppressor genes are epigenetic silenced during cancer initiation [47]. Thus, whole-genome hypomethylation and promoter hypermethylation causing a number of important ECM proteins down-regulated in cancer may provide new evidences for epigenetic control of tumor microenvironment on tumor progression.

## MATERIALS AND METHODS

### Cell culture

Human HCC cell line SMMC-7721 (TCHu13) was purchased from Cell Bank of the Chinese Academy of Sciences, and Huh7 (JCRB0403) from Japanese Collection of Research Bioresources. MHCC-97H cell line was obtained from Liver Cancer Institute, Zhongshan Hospital, Fudan University. Human liver cell line THLE-2 was purchased from American Type Culture Collection. Cells were cultured in Dulbecco's modified Eagle medium (DMEM) supplemented with 10% (v/v) fetal bovine serum (FBS) and 1% antibiotics at 37°C in a humidified incubator under 5% CO_2_ condition.

### Clinical tissue samples

All tissue samples were collected in Department of Liver Surgery, Renji Hospital, Shanghai Jiao Tong University School of Medicine. Fresh samples including tumor tissues and paracancerous liver (PCL) tissues were obtained from HCC patients during tumor resection. Two hunderd and two HCC samples were collected from 2004 to 2010 and were constructed into tissue microarray (TMA). The median age was 50 years (range 17-73 years). The majority of these patients were HBV-positive (187/202). The follow up was ended on December 2012, and the median period was 33 months (range 2-90 months). All tissue samples were obtained with informed consent and all procedures were performed in accordance with the China Ethical Review Committee.

### Immunohistochemical staining

All tissue samples were fixed in phosphate-buffered neutral formalin and routinely embedded in paraffin, and then cut into 4-μm-thick sections. The sections were detected with primary polyclonal antibody for DPT (Proteintech Group, Chicago, IL) overnight at 4°C. After incubated with the suitable second antibody, the sections were treated with diaminobenzidine and counterstained with haematoxylin. To quantify the level of DPT protein expression, all the sections were observed and photographed with a microscope (Carl Zeiss) and scored according to the ratio and intensity of positive-staining cells: 0-5% scored 0; 6-35% scored 1; 36-70% scored 2; 70-100% scored 3. The final expression level of DPT was designated as low and high group: score 0-1,low expression; 2-3, high expression. All the DPT expression level was quantified by two independent pathologists.

### Quantitative real-time PCR

The whole qPCR work was performed according to the MIQE guidelines (minimum information for publication of quantitative real-time PCR experiments) described by Bustin et al [48]. Total RNA was extracted from cells and tissues using Trizol reagent (Takara, Dalian, China) and purity and concentration of the isolated RNA were measured on NanoDrop ND-2000 spectrophotometer (Thermo Scientific, USA). Then RNA was reverse transcribed by PrimeScript RT Reagent kit (Takara, Dalian, China) according to the manufacturer's instruction in GeneAmp PCR System 9600 (Perkin Elmer, Norwalk, CT) at 37°C for 15 min and 85°C for 5 sec. The qPCR was subsequently performed with SYBR Premix Ex Taq (Takara) using an ABI7500 instrument (Applied Biosystems). 18s RNA was used as the reference gene for quantification, and relative standard curve was established for every qPCR assay. No template control (NTC) and reverse transcriptase minus control were negative for target gene DPT and reference gene 18s. Gene-specific primers used and the lengths of amplicons are listed in supplementary Table 2. Reactions were performed in triple in 20μl final volume reaction containing 4μl diluted cDNA, 20pmol/ul primers, 1×SYBR Premix PCR Master mix and 1×ROX. The running conditions were: denaturation at 95°C for 30 sec, followed by 40 cycles of PCR amplification including one step at 95°C for 15 sec and one step at 60°C for 31 sec (hybridization-elongation), and completed by melting curve stage at 68°C for 30 sec. The data were analyzed using the 2^−ΔCt^ approach.

### Western blotting

Whole cell lysates were prepared by lysis buffer (50mM Tris-HCl, 150mM NaCl, 1% Triton-X 100, 1mM MgCl_2_, MnCl_2_ and CaCl_2_, 1mM PMSF and 10mM sodium fluoride) [49]. The primary antibodies used included the following: DPT (HuaAn Biotechnology, Hangzhou, China), focal adhesion kinase (FAK) (Abcam, Cambridge, UK), p-FAK Tyr397 (Abcam, Cambridge, UK), Src (Cell Signaling Technology, Boston, MA), p-Src Tyr527 (Cell Signaling Technology, Boston, MA), ITGA3 (Abcam, Cambridge, UK), glyceraldehyde-3-phosphate dehydrogenase (GAPDH) (Proteintech Group, Chicago IL). After incubating with the IRDye 680 anti-mouse (LI-COR, Lincoln, NE) and IRDye 800 anti-rabbit (LI-COR, Lincoln, NE) secondary antibodies for 1 hour at room temperature, the bands were detected by an Odyssey infrared imaging system (LI-COR, Lincoln, NE). Quantification was analyzed by using Image J software.

### DAC and TSA treatment

Cells were treatment with 5 or 10μM of 5-aza-2′-deoxycytidine (DAC, Sigma-Aldrich, St. Louis, MO) or 300nM trichostatin A (TSA, Selleckchem, TX) for 3 days and drug in medium were replaced every 24 hours. Control cells were incubated with same volume DMSO. In drug combined treatment group, cells were cultured in the presence of 5μM of DAC for 2 days and were then treated for an additional 24 h with 300nM of TSA.

### Bisulfite sequencing

Genomic DNA (1μg) was bisulfite modified with a kit (EZ DNA Methylation-Gold ^TM^ Kit, Zymo Research, Orange, CA) according to the manufacturer's instruction. To amplify the CpG-rich regions of DPT promoter, we designed sequence-specific primers as follows: forward, 5′-TAGTTTAGGTTGGAGTGTAGTGG-3′; reverse, 5′-TAACTCATACTTATAATCCCAACAC-3′. The PCR products were purified and cloned into a vector pUC18-T (Sangon, Shanghai, China). The clones were selected through blue-white screening and finally clones which harbored the insert were sequenced.

### Subcutaneous injection

A total of 1.0×10^6^ of SMMC-7721/Lenti-vector or SMMC-7721/Lenti-DPT cells were implanted subcutaneously into the right flank of 5 male BALB/C nude mice in each group. Tumour sizes were measured once a week and mice were sacrificed at 6 weeks post-injection. Tumours were excised and their weights were determined. All procedures were performed in accordance with the East China Normal University Animal Care Commission.

### *In vivo* metastasis assays

The stable single cell clones of SMMC-7721 cells at 2×10^6^ infected with Lenti-DPT or Lenti-vector, were suspended in 40μl serum-free DMEM/matrigel (1:1) for each nude mouse. Through a 1-cm transverse incision in the upper abdomen under anesthesia, each nude mouse (7 in each group, 6 weeks male BALB/c-nu/nu) was orthotopically inoculated in the left hepatic lobe with a microsyringe. After 6 weeks, mice were sacrificed, and their livers were dissected, fixed with phosphate-buffered neutral formalin and prepared for standard histological examination. Mice were manipulated and housed according to protocols approved by the East China Normal University Animal Care Commission.

### Cells adhesion assays

Cells adhesion assays were performed as previously reported [50].

### Immunofluorescence cell staining

Assays were performed according to previous description [51]. Cells were incubated with vinculin (epitomics) or paxillin (epitomics) primary antibodies for 75 min at room temperature.

### GTPase pull-down assays and G-LISA assay

Cells were lysed and GTPase pull-down assays were performed according to procedures as described by Zhang et al. [50]. Activation of RhoA and Rac1 were measured using G-LISA activation assay kits (Cytoskeleton) according to the manufacturer's instructions. Cell extracts were added to 96-well plate coated Rho-GTP-binding protein. After incubation at 4°C for 30 minutes, the captured active, GTP-bound Rho GTPases were incubated with primary antibodies and detected with horseradish peroxidase (HRP)–conjugated secondary antibody.

### Statistical analysis

Data were presented as the means ± standard error of the mean (SEM). Statistical analyses were done using SPSS 19.0 for windows (IBM). Cumulative survival time was calculated by the Kaplan-Meier method and analyzed by the log-rank test. The chi-square test, or student's t-test were used for comparison between groups. Values of P < 0.05 were considered statistically significant.

More materials and methods were described in the supplementary data.

## Supplementary Materials and Methods






