# Supplementary material for: DNA methylation-mediated silencing of matricellular protein dermatopontin promotes hepatocellular carcinoma metastasis by α3β1 integrin-Rho GTPase signaling
Source: Oncotarget. 2014 Jul 21;5(16):6701–15. doi: 10.18632/oncotarget.2239 (PMC4196157; doi:10.18632/oncotarget.2239)
Supplement: Supplementary file 2 [file oncotarget-05-6701-s002.doc]

**Supplementary Table2**

qPCR target information: Description of the genes, primer/probes, amplicons, and other details

Supplementary Table 2A: Gene symbols, gene names, and accession numbers

| **Gene symbol** | **Gene name** | **Reference sequence**  **accession number** | **mRNA size**  **(bp)** |
| --- | --- | --- | --- |
| **DPT** | dermatopontin | NM_001937.4 | **1749** |
| **RNA18S5** | RNA, 18S ribosomal 5 | NR_003286.2 | **1869** |

Supplementary Table 2B: Measuring details of qPCR

| **Gene**  **symbol** | **Primer/probe sequences**  **(5’---3’)** | **Primer start in ref. sequence**  **(base no.)** | **Location of amplicon**  **(exon no.)** | **Intron spanning size (nt)** | **Amplicon**  **length (bp)** |
| --- | --- | --- | --- | --- | --- |
| **DPT** | F: 5’-TGACAGACAATGGAACTACG-3’  R: 5’-GACTCGAAGTAGCGGCTCTG-3’ | **F: 237 (Ex 1)**  **R: 376 (Ex 2)** | **Ex1/2** | **Ex1/2 = 14525** | 159 |
| **18s** | F: 5’-GTAACCCGTTGAACCCCATT-3’  R: 5’-CCATCCAATCGGTAGTAGCG-3’ | **F: 1577 (Ex 1)**  **R: 1708 (Ex 1)** | **Ex1** | **0** | **151** |
