# Supplementary material for: DNA methylation-mediated silencing of matricellular protein dermatopontin promotes hepatocellular carcinoma metastasis by α3β1 integrin-Rho GTPase signaling
Source: Oncotarget. 2014 Jul 21;5(16):6701–15. doi: 10.18632/oncotarget.2239 (PMC4196157; doi:10.18632/oncotarget.2239)
Supplement: Supplementary file 3 [file oncotarget-05-6701-s003.pdf]

DNA methylation-mediated silencing of matricellular protein dermatopontin promotes hepatocellular carcinoma metastasis by α3β1 integrin-Rho GTPase signaling

Supplementary Materials and Methods

Lentivirus production and cell transduction

The full-length cDNA encoding human DPT was amplified by PCR and cloned into pCDH-CMV-MCS-EF1-Puro vector (System Biosciences). Virus packaging was performed in 293T cells after cotransfection with DPT or mock vectors using Lipofectamine 2000 (Invitrogen, Carlsbad, CA). Viruses were harvested at 48 hour and 72 hour after transfection, and virus titers were determined. Target cells (1×10^5), including SMMC-7721 and Huh7 were infected with 1×10^6 recombinant lentivirus-transducing units in the presence of 6μg/ml polybrene (Sigma). The transfected cells were screened under 2μg/ml puromycin (Sangon, Shanghai) for 14 days. Puromycin resistant single-cell clones stably expressing DPT were established and verified by qPCR and Western blot analysis.

RNA interference-based gene knockdown experiment

For small interfering RNA (siRNA)-mediated ITGA3 silencing, the following target siRNA sequences were used: siRNA-1: 5’-CCCUGGCCAUCACAUUAUU-3’; siRNA-2: 5’-GCACCUUCAUCGAGGAUUA-3’; siRNA-3: 5’-GGAUGGAUUUCAGGAUAUU-3’; Control siRNA (scrambled): 5’-UUGGAGCGUGCGUAAGUAU-3’. The siRNA duplexes were designed and purchased from Eurogentec (Sart-Tilman, Belgium) and were transfected into cells according to the manufacturer’s instructions using the Lipofectamine RNAiMiX Reagent (Invitrogen, Carlsbad, CA).

Cell Proliferation Assay

Cells were seeded into a 96-well plate at 2×10^3 cells per well with 100 μl complete medium and cultured at 37°C. 10 μl Cell Counting Kit-8 (CCK-8, WST-8, Dojindo, Japan) solution was added to each well after 0 h, 24 h, 48 h, 72 h, 96 h and 120 h, respectively. In viable cells, WST-8 was metabolized to produce a colorimetric dye that was detected at 450 nm using a microplate reader. The experiment was performed in triplicate and repeated triple.

Soft agar colony formation assays

Stable overexpressing DPT cell lines SMMC-7721, Huh7 and their control cells were suspended and 2×10^3 cells were planted on a 6cm plate containing 0.6% base agar and 0.35% top agar. Cells were incubated at 37°C for 21 days. Then, clones were stained with 0.005% crystal violet and all the visible colonies were counted under microscope. The experiments were repeated at least 3 times.

***In vitro* migration and invasion assays**

SMMC-7721 or Huh7 cells were detached and resuspended in serum-free DMEM. For migration assay, approximately 5×10^4 cells in 0.2 ml were placed into the upper chamber of a transwell (Millipore) and incubated for 24h. For invasion assay, approximately 1×10^5 cells in 0.1 ml were placed in Matrigel (BD biosciences, Bedford, MA)-coated inserts (Millipore) seated on the 24-well plate. DMEM containing 10% (v/v) FBS was added to the bottom chamber. Cells were incubated at 37°C and allowed to invade through Matrigel for 48 h. In recombinant DPT treatment assay, recombinant human DPT (R&D Systems, Minneapolis, MN) were added to the medium for the final concentration at 5，10 or 20μg/ml. For ITGA3 blocking, cell suspensions in serum-free DMEM (after siRNA transfection) were added to the upper inserts. After incubation, filters were fixed and stained with 0.1% (w/v) Crystal Violet. Non-invading cells

were removed using a cotton swab while invading cells on the underside of the filter were counted under a microscope at a magnification of 200× or 400×. At least six grids per field were counted and the experiments were repeated at least twice.

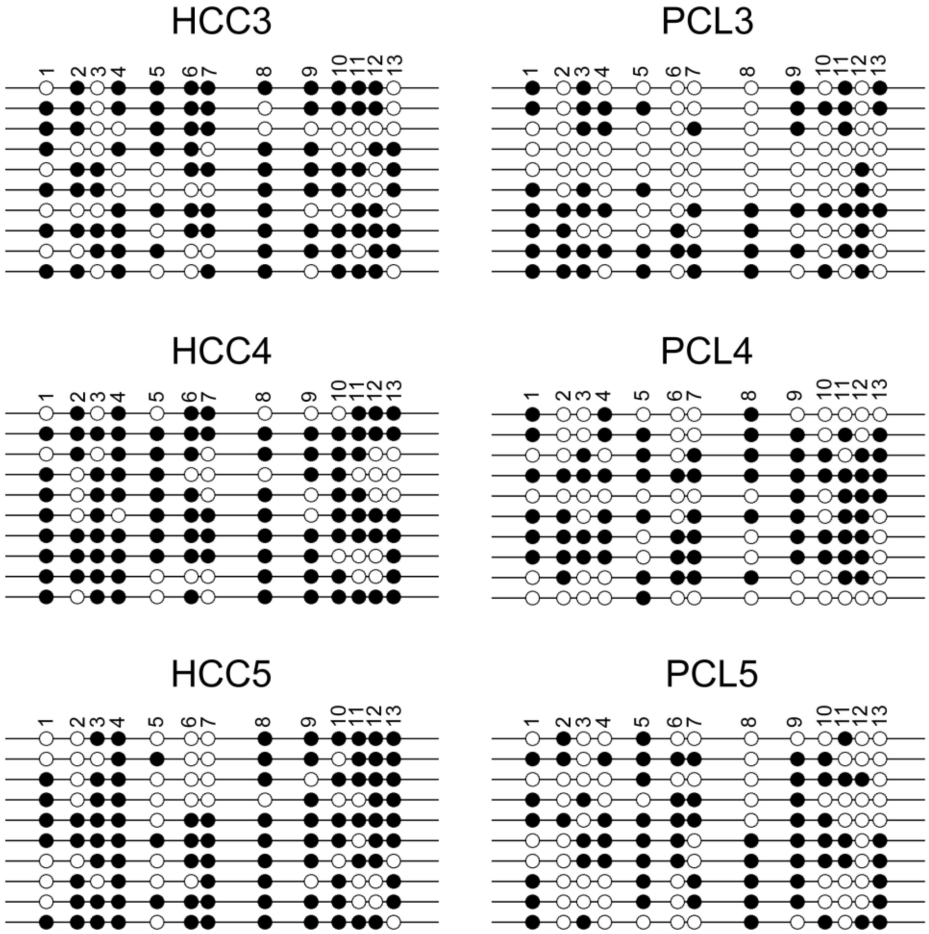

Supplementary Figure1: Bisulfite-sequencing results of other 3 pairs of HCC and their paracancerous liver (PCL) tissues. Open circles indicate unmethylated and solid circles represent methylated CpG dinucleotides.

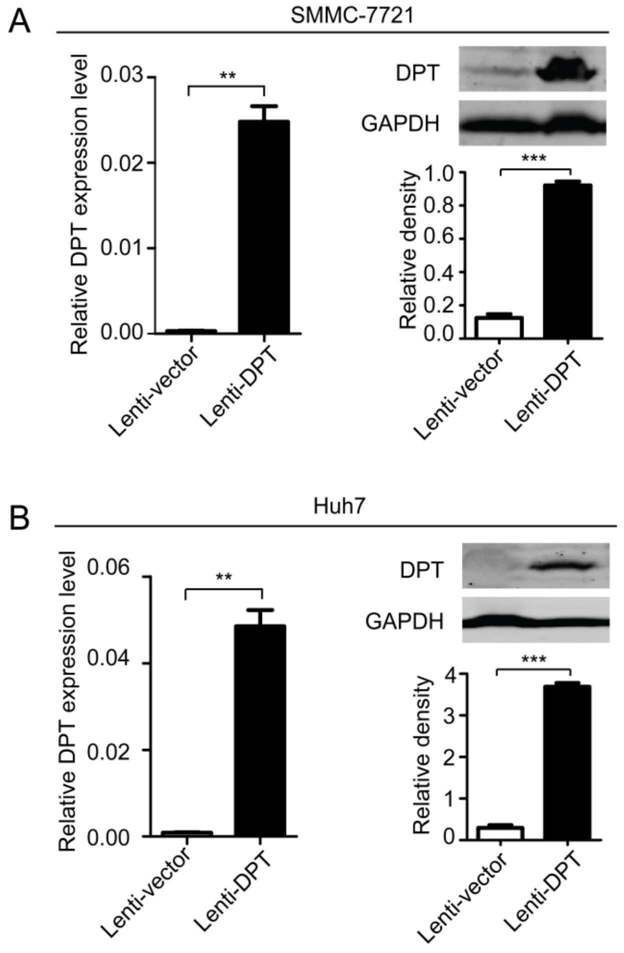

Supplementary Figure2: Verification of DPT overexpression in SMMC-7721 and Huh7 cells. (A) The overexpression of DPT was validated in SMMC-7721 cells using qPCR (left panel) and western blotting analysis (right panel). (B) The overexpression of the DPT mRNA and protein was detected in Huh7 cells. Values are means ± SEM (***P* < 0.01, *** P < 0.001).

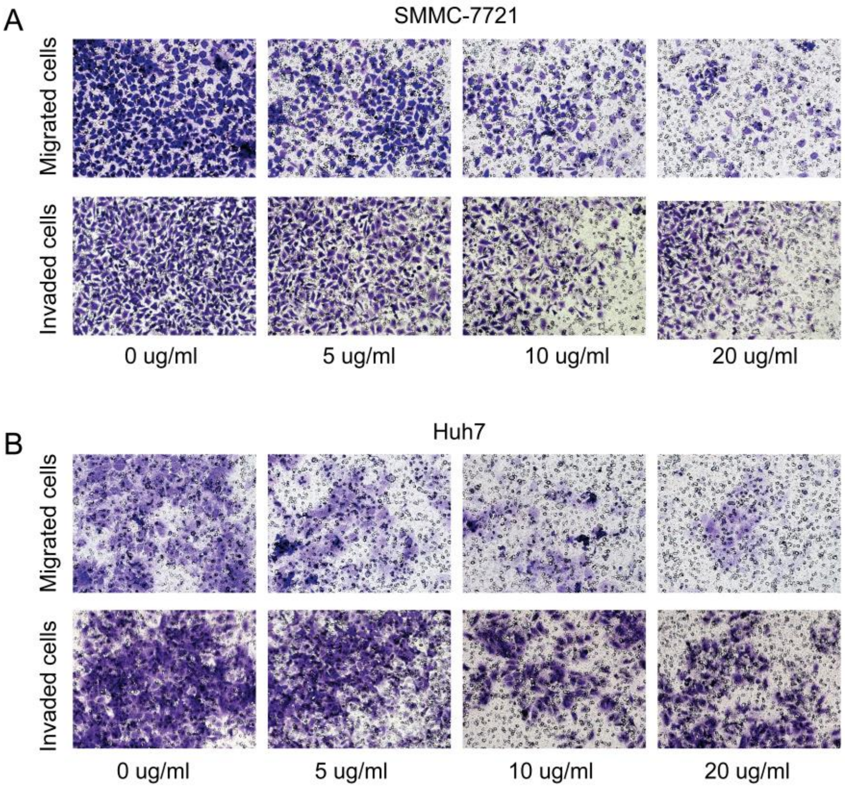

Supplementary Figure3: Recombinant human DPT suppresses SMMC-7721 and Huh7 cells migration and invasion *in vitro*. (A) Representative photographs of migrated and invaded SMMC-7721 cells treated with 0，5，10 or 20 μg/ml recombinant human DPT. (B) Representative photographs of migrated and invaded Huh7 cells treated with different concentration of recombinant human DPT.
